# Editorial: Natural Plant Antioxidants and Cardiovascular Disease

**DOI:** 10.3389/fphys.2022.848497

**Published:** 2022-02-16

**Authors:** Claudia Castro

**Affiliations:** Consejo Nacional de Investigaciones Científicas y Técnicas (CONICET), Instituto de Medicina y Biología Experimental de Cuyo (IMBECU) and Universidad Nacional de Cuyo, Facultad de Ciencias Médicas, Instituto de Bioquímica y Biotecnología, Mendoza, Argentina

**Keywords:** native plants, vascular biology, cell proliferation, atheroma, cell signal

According to the World Health Organization (WHO), cardiovascular disorders are one of the main causes of death worldwide. The prognosis and the mortality rate are directly related to the vascular damage favoring the progression toward heart failure. One pathogenic mechanism and common nexus of chronic pathologies and chronic inflammation is oxidative stress characterized by a biochemical imbalance between the production of reactive oxygen species (ROS) and the endogenous antioxidant defense system. In metabolic process, oxygen undergoes a series of reduction steps leading to the production of free radicals represented by Superoxide (O·-2), Oxygen radical (O··2), Hydroxyl (OH·), Alkoxyradical (RO·), and Peroxyl radical (ROO·). Under physiological conditions, small quantities of ROS are intracellular produced which act in cell signaling, and can be readily reduced by the antioxidant defense system. The unbalance between the oxidant species and the antioxidant compounds may increase harmful species that induce structure modification and functional modulation of proteins, lipids and nucleic acids. These alterations disturb homeostasis of the organism due to the damage produced at cellular, tissue, and systemic levels. Oxidative damage has been implicated in the etiology or pathology of cardiovascular diseases including endothelial dysfunction, atherosclerosis, hypertension, heart failure, and is involved in metabolic syndrome and obesity, responsible for a rise in stroke incidence among young adults. On vascular wall, ROS modulate cell growth, apoptosis, migration, inflammation, secretion, and extracellular matrix production, acting through various mechanisms: (1) damaging direct action on the endothelium and the vascular smooth muscle cells; (2) decrease in nitric oxide bioavailability due to oxidative inactivation; (3) formation of peroxynitrite, a radical with powerful lipid oxidizing and constricting action; (4) changes in the endothelial metabolism of eicosanoids (molecules involved in various signaling pathways related to inflammatory processes) and (5) oxidative modifications of LDL.

The properties of plant products as antioxidants have been of worldwide interest. Since ancient times, they have been used to mitigate or cure diseases and the analysis of the ability of their components to counteract oxidative stress has been increasing noticeably.

In plant organisms, evolution has defined and selected numerous strategies to defense, attack, adapt and communicate, generating an immense number of bioactive molecules. Given their wide structural and biological diversity, natural products (NP) represent the most abundant source of identified chemical structures, making them ideal as a basis for the rational design of drugs. In this context, NP chemistry plays an especially important role as the majority of drugs currently approved for human use (60%) are being isolated or derived from NP. In recent years, the study of the beneficial effect on human health of the consumption of antioxidants contained in NP has become very relevant. Among the components of these products with greater antioxidant capacity are polyphenols and flavonoids. There are more than thousands of species for which folk medicine describes different uses in the care and preservation of health. Studies have been carried out on the potential properties of the flora, leaves, roots, and their aqueous and/or ethanolic extracts, describing antioxidant, anti-lipidic, anti-atherosclerotic, anti-glycemic, and anti-mitogenic effects.

The Research Topic present in this issue offers the opportunity to revise the importance of compounds derivate from natural plants with antioxidant and anti-inflammatory properties on cardiovascular diseases. The main goal is to offer a collection of high quality papers that increase the knowledge about the effect of bioactive compounds present in plants on vascular biology.

In this collection you would find an interesting review about the influence of flavonoids on the gut microbiota, and the mechanism of new bioactive molecules derived from this interaction, in the occurrence and development of atherosclerosis, myocardial infarction, heart failure, and hypertension. Flavonoids show a potential value in the prevention and treatment of cardiovascular disease following their transformation through gut microbiota metabolism.

In order to provide new ideas for the prevention and treatment of cardiovascular diseases, the molecular mechanisms of different antioxidants, extracted from native plants, are included in this Research Topic. First, novel data of anti-restenotic properties of Jujuboside B (JB), one of the main biologically active ingredients extracted from the seeds of Zizyphus jujube, is presented. JB antagonizes PDGF-BB-induced phenotypic switch, proliferation and migration of vascular smooth muscle cells partly through AMPK/PPAR-γ pathway.

Then, cardioprotective therapy is presented with a strong antioxidant extracted from Chinese herbal medicine, the astragalus, called astragaloside IV (ASIV), which showed the ability to alleviate myocardial ischemia-reperfusion (I/R) injury, caused by autophagosome accumulation. Finally, new data is presented about the beneficial effects of two South American origin shrubs, Prosopis strombulifera (Ps), and Tessaria absinthioides (Ta). Consumption of aqueous extract of Ps or Ta produces anti-oxidant/anti-mitogenic/anti-atherosclerotic effects and the use of these plants may be beneficial as a complementary strategy regarding cardiovascular therapies.

The increasingly frequent use of phytochemicals determines the importance of running studies with the purpose of evaluating their properties, which facilitates the enrichment of the cultural interpretation of the popular use of different plants when investigating possible biological effects. The use of natural antioxidants for the treatment of different pathologies is based on a long popular tradition, with empirical evidence, but with little scientific backup to support it from the point of view of basic or clinical studies. The study of the traditional use of medicinal plants applying biological tests provides information, with scientific bases, about the efficacy and safety of potential therapeutic agents obtained from plants.

## Author Contributions

The author confirms being the sole contributor of this work and has approved it for publication.

## Conflict of Interest

The author declares that the research was conducted in the absence of any commercial or financial relationships that could be construed as a potential conflict of interest.

## Publisher's Note

All claims expressed in this article are solely those of the authors and do not necessarily represent those of their affiliated organizations, or those of the publisher, the editors and the reviewers. Any product that may be evaluated in this article, or claim that may be made by its manufacturer, is not guaranteed or endorsed by the publisher.

